# The Effects of Alcohol Hangover on Executive Functions

**DOI:** 10.3390/jcm9041148

**Published:** 2020-04-17

**Authors:** Craig Gunn, Graeme Fairchild, Joris C. Verster, Sally Adams

**Affiliations:** 1 Addiction and Mental Health Group, Department of Psychology, University of Bath, Bath BA2 7AY, UK; 2 Department of Psychology, University of Bath, Bath BA2 7AY, UK; G.Fairchild@bath.ac.uk; 3 Division of Pharmacology, Utrecht University, 3584CG Utrecht, The Netherlands; J.C.Verster@uu.nl; 4 Institute for Risk Assessment Sciences (IRAS), Utrecht University, 3584CM Utrecht, The Netherlands; 5 Centre for Human Psychopharmacology, Swinburne University, Melbourne, VIC 3122, Australia

**Keywords:** alcohol, hangover, executive functions, working memory, cognition

## Abstract

Recent research has suggested that processes reliant on executive functions are impaired by an alcohol hangover, yet few studies have investigated the effect of hangovers on core executive function processes. Therefore, the current study investigated the effect of hangovers on the three core components of the unity/diversity model of executive functions: the ability to switch attention, update information in working memory, and maintain goals. Thirty-five 18-to-30-year-old non-smoking individuals who reported experiencing a hangover at least once in the previous month participated in this study. They completed tasks measuring switching (number-switching task), updating (n-back task), and goal maintenance (AX Continuous Performance Test, AX-CPT) whilst experiencing a hangover and without a hangover in a ‘naturalistic’ within-subjects crossover design. Participants made more errors in the switching task (*p* = 0.019), more errors in both the 1- (*p* < 0.001) and 2-back (*p* < 0.001) versions of the n-back, and more errors in the AX-CPT (*p* = 0.007) tasks when experiencing a hangover, compared to the no-hangover condition. These results suggest that an alcohol hangover impairs core executive function processes that are important for everyday behaviours, such as decision-making, planning, and mental flexibility.

## 1. Introduction

An alcohol hangover is a combination of mental and physical symptoms, experienced the day after a single episode of heavy drinking, when blood alcohol concentration (BAC) approaches zero [1]. It is the most common negative consequence of heavy drinking and can impair cognitive processes, such as sustained attention, memory, and psychomotor skills [2,3]. However, relatively few studies have investigated the effect of alcohol hangovers on core components of executive functions.

Executive functions are higher-order cognitive processes used in everyday behaviours, such as decision-making, mental flexibility, and planning. Recent studies have indicated that executive functions may be negatively influenced by alcohol hangovers. Studies have suggested that performance on tasks of interference control [4,5] and response inhibition [6] is impaired when subjects are experiencing a hangover, suggesting poorer inhibitory control, which may negatively influence decisions around subsequent alcohol use [7] and emotion regulation [8]. Furthermore, findings showing poorer spatial working memory [4], reward learning [9], prospective memory [10,11], semantic verbal fluency [10], and performance on backward visual span tasks [12] indicate that executive functions are impaired whilst experiencing a hangover. A recent report by the Institute of Alcohol Studies suggested that the cost of hangover-related reductions in work productivity could be as high as £1.4 billion per annum in the UK [13]. As effective workplace performance relies on an individual’s ability to make decisions, organise tasks, and plan, detrimental effects of hangovers on executive functions may contribute toward these costs. Therefore, it is important to understand how these processes may be influenced the morning after a night of heavy alcohol consumption, i.e., during a hangover.

Executive functions are utilised when behaviours need to be controlled (rather than when they are ‘automatic’), when cognitive processes are combined, or when individuals need to switch attention between tasks [14]. The unity/diversity model conceptualises executive functions as being composed of two core components, alongside a single common factor that is utilised in all executive function tasks [15]. The two components represent the ability to switch attention from one task/mental set to another (switching) and the ability to update information within working memory (updating). The common factor of the unity/diversity model represents the ability to maintain and manage goals, in order to effectively complete tasks (goal maintenance). All executive function tasks utilise aspects of these core components. As hangover-related impairments have been observed in higher-order cognitive processes, such as prospective memory [10], it is possible that hangovers influence these core components of executive function.

Attentional switching requires allocation of attentional resources to effectively switch from one task or mental set to another [16]. Recent studies have indicated that a hangover may be a state in which individuals experience high cognitive load [17] and thus have fewer available resources to switch attention [18,19]. When available cognitive resources are low, completion of executive function tasks becomes ineffective or inefficient [20,21,22]. Factors associated with heavy alcohol consumption, such as a reduction in glutamatergic and an increase in GABAergic, dopaminergic, and serotonergic neurotransmission, may also influence attentional switching [23,24]. During hangover, dopaminergic neurotransmission may be reduced, and noradrenaline may be elevated [9,25], suggesting that switching could become impaired. Furthermore, studies have highlighted that fatigue (which is one of the most commonly reported symptoms of a hangover [26]) can lead to impairments in switching [27].

Thus far, studies investigating attentional switching in individuals experiencing a hangover have yielded mixed results. One study induced hangovers experimentally [19] and reported no effect on switch costs, reflecting the additional time needed to switch attention to the new rule set. However, experimental hangover manipulations involve administering lower doses of alcohol than are typically consumed when drinking in everyday life [28], and this practice could influence the effects of a hangover [29]. Two naturalistic studies, which involve assessing the impact of hangovers experienced following real-life drinking, investigated the effects of a hangover on perseveration errors, which are erroneous responses made according to the previously correct rule or set, reflecting a switching failure. One reported that a hangover did not influence switching performance in a non-student sample [4], whereas another study using a student sample indicated poorer switching accuracy when experiencing a hangover, as compared to a control condition [30]. It is possible that hungover individuals attempt to maintain performance on switching tasks by either sacrificing accuracy to maintain the speed of their responses or by sacrificing speed to maintain accuracy (i.e., a ‘speed–accuracy trade-off’).

To our knowledge, no studies have investigated the effects of an alcohol hangover on updating or goal maintenance; however, there are indications that both processes could be negatively affected by a hangover. Goal maintenance is an important process utilised to complete all executive function tasks [15]. For example, an individual completing a task at work (e.g., writing a report) would need to keep his or her overall goal in mind whilst planning, organising, and making decisions about the individual task subcomponents. If goal maintenance is impaired, individuals may be less effective or efficient at completing complex tasks with multiple subcomponents. As previously mentioned, studies have indicated impairments in working memory performance, prospective memory, and semantic verbal fluency—all tasks requiring executive functions—during a hangover [4,10,11,12]. Therefore, it is possible that a common factor underlying hangover-related impairments in each of these tasks is a deficit in the ability to maintain goals. Inhibitory control is also impaired when experiencing a hangover [4,5,6] and is a key part of goal maintenance [15], further suggesting goal maintenance could be influenced by a hangover. In addition, reduced cognitive resources during a hangover may influence goal maintenance by biasing individuals toward reacting to external events (i.e., bottom-up stimulus-driven processing) rather than proactive control of one’s actions (i.e., actively sustaining goal representations through top-down processing) [18,31]. The AX Continuous Performance Task (AX-CPT) can be used to assess goal maintenance and can differentiate between proactive and reactive control [32].

The process of updating information in working memory can become impaired by high cognitive load and when there is a reduction in available cognitive resources [33]. As previously mentioned, cognitive resources may be reduced during a hangover [18,19], negatively affecting the ability to update information in working memory. Furthermore, a study of the cognitive effects of pain indicate that a headache can impair performances on tasks measuring updating [34]. By using an n-back task with conditions that vary in difficulty, studies have also demonstrated that cognitive load selectively influenced the disrupting effect of pain on updating [35]. In addition, studies have indicated that updating can be impaired following sleep deprivation [36]. As a headache is a ‘core’ hangover symptom [1], and individuals experience sleep disruptions after heavy alcohol consumption (e.g., decreased efficiency and REM sleep, increased night-time awakenings [37], and decreased sleep duration [38]), updating ability may also be compromised by a hangover. To assess this possibility, we used an n-back working memory task with two conditions that vary in difficulty (1-back and 2-back).

In summary, the current study aimed to investigate the effects of an alcohol hangover on all three core components of the unity/diversity model of executive functions: switching, updating, and goal maintenance. Specifically, we hypothesised that participants experiencing a hangover would show impairments in: (1) switching, (2) updating, and (3) goal maintenance, as compared to the no-hangover control condition. We also hypothesised that participants would adopt a more reactive control style on the AX-CPT task in the hangover condition, as compared to the no-hangover condition, and that the magnitude of impairments in goal maintenance, updating, and switching abilities would be positively associated with hangover severity. As performance on executive function tasks may be related to an individual’s confidence in his or her ability to complete tasks (self-efficacy: the belief we have in our ability to execute the actions required for specifically designated performances, usually assessed as our degree of confidence that we can perform specific tasks [39]), and self-efficacy to complete tasks is lower when individuals are experiencing a hangover [12,40], we also explored the relationship between self-efficacy and task performance. We hypothesised that performance in goal maintenance, updating, and switching tasks would be positively associated with self-efficacy to complete these tasks.

## 2. Materials and Methods

### 2.1. Participants

Thirty-eight participants were recruited from a student population by poster/flyer and digital advertisements, the University of Bath’s research participation scheme, word of mouth, and direct approach by the researcher. Inclusion criteria required participants to consume at least 6 (female) or 8 (male) units of alcohol in a typical heavy drinking session, to be aged between 18 and 30 years old, to be non-smokers, and to be in general good mental and physical health. To exclude the potential confound of hangover-resistance, only participants who reported experiencing a hangover in the past month were recruited. Participants who were pregnant/breast-feeding, taking medication or recreational drugs, consuming > 400 mg of caffeine per day, had a current or past personal or family history of drug dependency, or had a diagnosed sleep disorder were excluded. Three participants withdrew before completing both conditions; thus, 35 participants (14 males; 21 females) completed the study. The University of Bath Psychology research ethics committee approved this research, ethics code: 18-328.

### 2.2. Design

An experimental ‘naturalistic’ design, with one within-subjects factor of condition (hangover and no-hangover) was used. The naturalistic design is a valid method when one is interested in examining the real-life cognitive effects of alcohol hangover, and it has been successfully implemented in many hangover studies [41]. The hangover condition took place on a morning following an evening of heavy alcohol consumption, and the no-hangover condition on a morning following no alcohol consumption for at least 24 h prior to testing. Order of testing was counterbalanced across subjects, whereby 53% of participants completed the hangover condition first.

### 2.3. Measures

Participants completed three cognitive tasks assessing different components of executive function: switching, updating, and goal maintenance.

#### 2.3.1. Number-Switching Task

**Figure 1:**
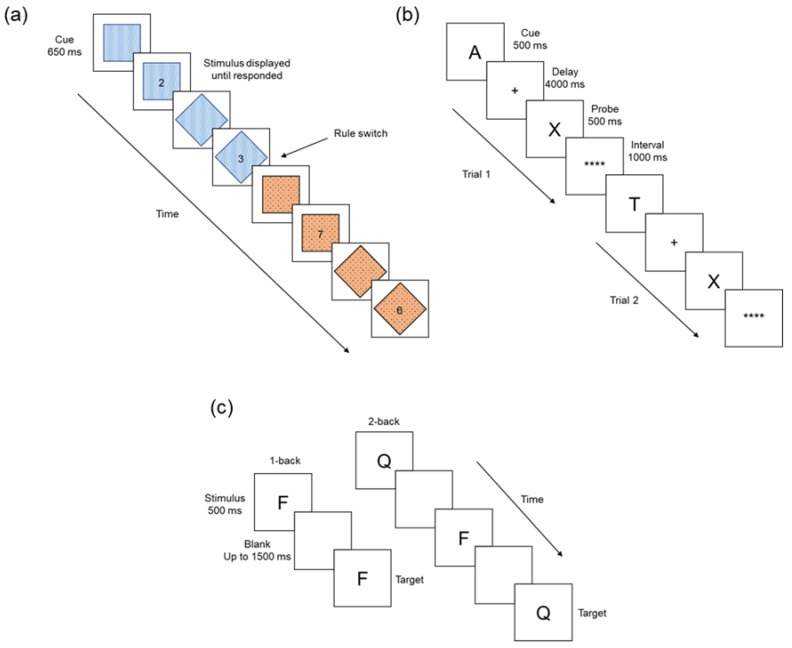
Schematic representations of each cognitive task. (**a**) In the switching task, participants are presented with a cue (empty blue/orange shape), followed by a number stimulus. Participants respond according to the rule (determine odd/even or higher/lower than 5), indicated by the colour of the shape (blue or orange). (**b**) In the AX Continuous Performance Task (AX-CPT) task, participants are presented with a cue-probe pair separated by a long delay (+). When **** appeared on the screen, participants respond by pressing the ‘m’ key when the cue is ‘A’ and probe is ‘X’; otherwise, participants respond with the ‘z’ key. The first trial is an example of a target trial (AX) and the second trial is an example of a BX non-target trial type (the cue “T” is incorrect in this case). (**c**) In the n-back task, participants respond with the ‘m’ key when the target is the same as the stimulus presented either 1 or 2 trials earlier (e.g., if the target is the same as the previous letter in the 1-back version); otherwise, participants respond with the ‘z’ key.

A cued-switching task was used to measure switching [42]. In this task, participants were presented with a string of numbers (1, 2, 3, 4, 6, 7, 8, and 9) appearing within a shape (square or diamond). A cue (square/diamond without number) appeared for 650 ms before the number stimulus. Participants were instructed to respond depending on the ‘rule’, which was indicated by the colour of the shape. Participants responded with ‘z’ if the number was odd or ‘x’ if the number was even, when presented within a blue shape, and responded with ‘n’ if the number was lower than 5 or ‘m’ if the number was higher than 5, when presented within an orange shape. The rule was switched every 4 trials, in a sequential manner. The primary outcome measures were switch costs, which were calculated by subtracting RT for the second trial following a rule change (P2) from the first trial following a rule change (P1) and perseveration errors, i.e., erroneous responses made according to the prior rule set. Schematic representations of each task are presented in Figure 1.

#### 2.3.2. The N-Back Task

The letter version of the n-back task was used to measure updating [43]. In this task, participants viewed a string of letters (random presentation) and were asked to indicate whether the letter was the same as the letter presented in a previous trial (i.e., n-back). Letters were presented for 500 ms, with an inter-trial interval (blank screen) for 1500 ms. Participants were asked to respond with ‘m’ when the letter was the same as n-back (target trials), and ‘z’ when it was not the same (non-target trials). The task consisted of two 1-back (letter same as the previous trial) and two 2-back (letter same as the one presented before the last trial) blocks presented in alternating blocks (i.e., 1-back, 2-back, 1-back, and 2-back). There were 45 trials in each block, with target stimuli (those that are valid n-back trials) presented 33% of the time. The primary outcome measure for this task was errors to target stimuli.

#### 2.3.3. The AX Continuous Performance Task

The AX Continuous Performance Task (AX-CPT) can be used to assess goal maintenance and can differentiate between proactive and reactive aspects of cognitive control [32,44,45]. Participants respond to a probe on the basis of a preceding cue. A letter cue was presented on screen for 500 ms, followed by a long delay of 4000 ms (displayed as ‘+’) [32]. Participants were then presented with a letter probe for 500 ms, followed by an inter-trial interval of 1000 ms (displayed as ‘****’). Participants responded to probes by pressing ‘m’ on the keyboard for cue-probe targets or ‘z’ for non-targets. Target responses are when an ‘A’ cue is followed by an ‘X’ probe (AX-type trial), and non-target trials are responses to all other letter sequences. ‘AY-type’ trials are when an ‘A’ cue is followed by any probe other than ‘X’; ‘BX-type’ trials are those when any cue other than ‘A’ are followed by an ‘X’ probe; and ‘BY-type’ trials occur when any cue other than ‘A’ is followed by any probe other than ‘X’. Target trials (AX) were presented with 70% frequency, and non-targets with 30% frequency; non-target trial frequency was equally distributed so that non-cue–probe (e.g., BX-type), cue–non-probe (e.g., AY-type), and non-cue–non-probe (e.g., BY-type) trials each occurred 10% of the time. A total of 120 trials were presented in a single block, and the primary outcome measure was the number of errors for each trial type. Participants utilising reactive control selectively retrieve contextual information when stimuli are presented, and they are less likely to actively maintain contextual information. In the AX-CPT task, reactive control can be observed with increased errors in ‘BX-type’ trials as participants react to a valid stimulus (the ‘X’), but without actively maintaining the preceding invalid cue (not an ‘A’). Thus, if individuals with a hangover are biased toward reactive control processes, we would expect to observe an increase in erroneous responses to ‘BX-type’ trials relative to the no-hangover control condition.

#### 2.3.4. Subjective Measures

Self-reported alcohol consumption on the previous night was used to calculate estimated peak BAC (eBAC), using the Widmark formula [46]. Hangover severity was measured by using a 1-item hangover severity scale and modified Alcohol Hangover Severity Scale (mAHSS; [47]). Participants were also asked to rate how confident they felt about completing the tasks effectively (self-efficacy) on an 11-point scale (0 = cannot do at all; 10 = certainly can do; [39]), following practice trials on each cognitive task. Following each task, participants were asked to complete the Rating Scale of Mental Effort (RSME) which assessed the degree of effort involved in performing the respective task [48].

### 2.4. Procedure

Participants were given information about the study and were booked in for two sessions (hangover and no-hangover), according to when they next expected to experience a hangover or have a no-hangover day. Time of day of testing was as similar as possible for both sessions. Participants were screened to ensure they met inclusion criteria and gave written informed consent before the study started. Participants self-reported their previous night’s alcohol consumption by using pictorial prompts labelled with alcohol unit content and caffeine consumption on the day of testing. Participants were breathalysed and completed the 1-item hangover severity scale and mAHSS, to verify their condition (hangover and no-hangover) before completing the three cognitive tasks in a randomised counterbalanced order. Following practice trials, participants rated their self-efficacy before completing each task. Following completion of each task, participants completed the RSME. Participants then arranged the second testing session at least 36 h later, to prevent crossover effects. Upon completion of both conditions, participants were paid £10 and received a full debrief.

### 2.5. Statistical Analysis

Statistical analysis was conducted in accordance with our preregistered protocol [49]. Outliers were removed if they were > 1.5 * Inter-Quartile Range and > 2 SD from the mean. Analysis was also conducted by winsorizing the outliers, which did not impact the results presented below. For the switching task, trials following an error and trials with RT > 2500 ms were omitted from analysis. Participants for whom < 50% trials were available were removed from analysis (*n* = 5). Error trials were omitted from RT analysis [50]. Repeated measures ANOVAs were conducted with order and sex as between-subject factors, using SPSS (version 25). Effect sizes are reported as Cohen’s *d*. Due to the possible effects of acute intoxication at BAC > 0.02% [51], a sensitivity analysis was conducted to see if residual alcohol concentrations during a hangover influenced cognitive performance. A sensitivity analysis, excluding one participant with a BAC > 0.02%, yielded similar results; therefore, this participant is included in the analyses presented below.

## 3. Results

### 3.1. Participant Characteristics

**Figure 2:**
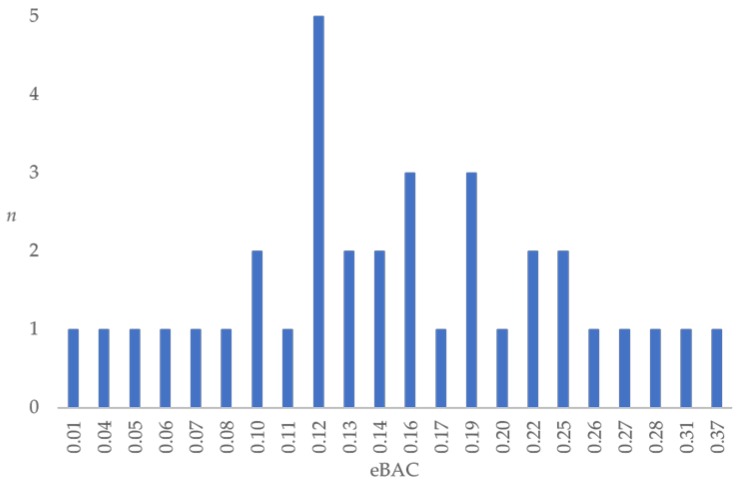
A visual representation of the range of eBAC values during the drinking episode preceding the hangover condition and number of participants experiencing each eBAC value.

The average age of participants was 20.23 years (SD = 2.81; range = 18–30), and they consumed an average of 13.28 alcohol units the evening before the hangover condition (SD = 5.13; range = 5–28.5). The mean eBAC calculated for the evening before the hangover condition was 0.16% (SD = 0.08; range = 0.01%–0.37%). None of the participants consumed alcohol before the no-hangover control condition or reported experiencing a hangover (i.e., all participants scored zero on the hangover-severity scale). Although eBAC calculations for some participants were low (e.g., 0.01%), all participants in the hangover condition reported having a hangover (severity scale score > 0). A sensitivity analysis indicated that excluding participants with an eBAC < 0.05% the night before the hangover condition did not alter results, and these participants were therefore included in the analyses reported below. A visual representation of the range of eBAC values in the sample is provided in Figure 2. There was no difference in caffeine consumption between the hangover and no-hangover conditions (*p* = 0.781).

### 3.2. Effects of Hangover on Switching

**Figure 3:**
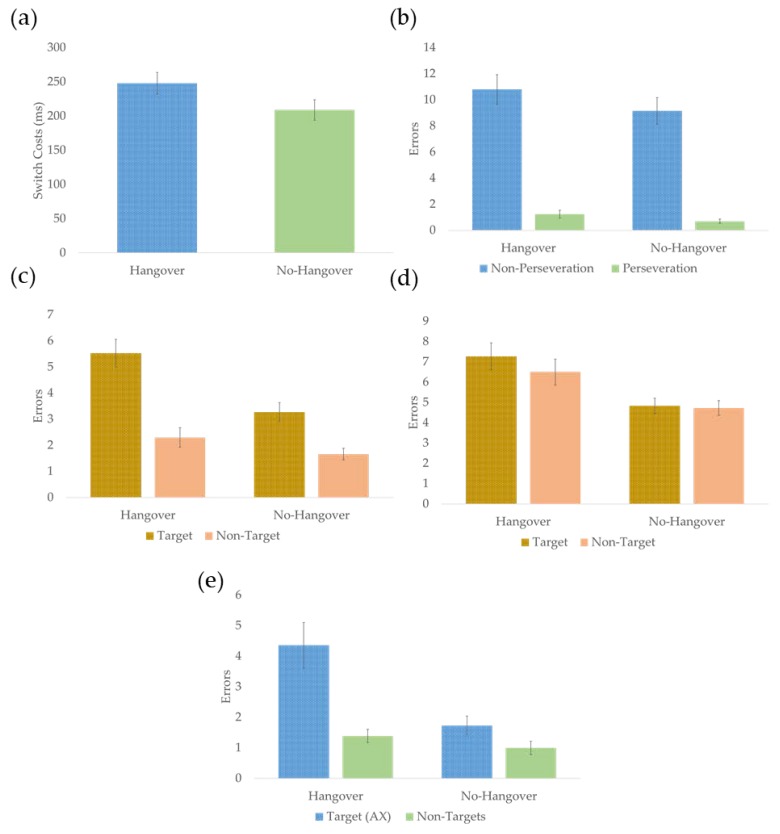
Graphical representations of the main effects from the three cognitive tasks. (**a**) Relative to the no-hangover condition, mean switch costs on the switching task tended to be greater when individuals were experiencing a hangover. (**b**) Relative to the no-hangover condition, mean errors on the switching task were higher when individuals were experiencing a hangover. (**c**) Relative to the no-hangover condition, errors for non-target and target stimuli in the 1-back version of the n-back task were greater in the hangover condition. (**d**) Relative to the no-hangover condition, errors in the 2-back task were greater overall in the hangover condition. (**e**) Relative to the no-hangover condition, errors on AX trials of the AX-CPT task were greater in the hangover condition. The error bars represent ±1 standard error of the mean.

**Figure 4:**
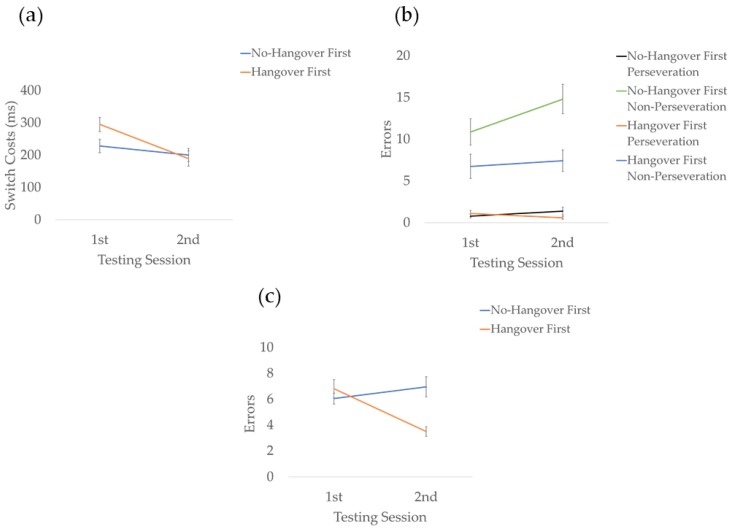
Graphical representations of the condition * order interactions. (**a**) Switching speed decreased (lower switch costs) across testing days when the first testing session was the hangover condition, but not when the first testing session was the no-hangover condition. (**b**) Switching accuracy declined (greater number of non-perseveration errors) across testing days when the first testing session was the no-hangover condition, but not when the first testing session was the hangover condition. (**c**) Updating performance improved (fewer errors) across the testing days for those completing the hangover condition first, but not for those completing the no-hangover condition first. The error bars represent ±1 standard error of the mean.

For reaction times, the analysis of switch costs indicated a trend-level main effect for condition (*F* (1, 26) = 3.359, *p* = 0.078, *d* = 0.72), whereby switch costs were marginally greater in the hangover relative to the no-hangover condition. There was also a condition *order interaction (F (1, 26) = 9.850, *p* = 0.004, *d* = 1.23) indicating performance improved (lower switch costs) across testing days when the first testing session was the hangover condition (*F* (1, 26) = 13.748, *p* = 0.001, *d* = 1.45). However, there were no significant differences between testing days for those who completed the task for the second, time when hungover (*p* = 0.387). There were no other significant effects or interactions. Results for main effects on each task are presented graphically in Figure 3, condition * order interactions are presented in Figure 4, and means and SDs are presented in Table 1.

For errors, analysis indicated a main effect of condition (*F* (1, 22) = 6.392, *p* = 0.019, *d* = 1.08), whereby errors were greater overall in the hangover relative to no-hangover condition. There was also a main effect of error type (*F* (1, 26) = 77.544, *p* < 0.001, *d* = 3.75), whereby there was a greater number of non-perseveration than perseveration errors. An order * error-type interaction indicated non-perseveration errors were greater for those who completed the no-hangover condition first than those who completed the hangover condition first (*F* (1, 22) = 8.301, *p* = 0.009, *d* = 1.23). There was also a greater number of non-perseveration errors than perseveration errors in both orders of condition (*p*s < 0.001). A condition * order interaction (*F* (1, 26) = 7.483, *p* = 0.012, *d* = 1.17) indicated that performance significantly declined across testing days when the first testing session was the no-hangover condition (F (1, 22) = 11.650, *p* = 0.002, *d* = 1.45), whereas there were no significant differences between testing days for those who completed the task for the second time, when sober (*p* = 0.872). The analysis also indicated that participants who were hungover during their second session made greater errors in the hangover condition than those who were hungover during their first session (F (1, 22) = 12.958, *p* = 0.002, *d* = 1.54). A condition * order * error-type interaction indicated that order effects were restricted to non-perseveration errors (*F* (1, 26) = 6.428, *p* = 0.019, *d* = 1.08) (see Figure 4b). There were no other significant effects or interactions.

### 3.3. Effects of Hangover on Updating

To investigate the effect of a hangover on updating, each version of the n-back task was analysed separately. For the 1-back version, there was a main effect of condition (*F* (1, 31) = 20.734, *p* < 0.001, *d* = 1.64), whereby errors were greater in the hangover than the no-hangover condition. There was also a main effect of trial type (*F* (1, 31) = 25.399, *p* < 0.001, *d* = 1.81), whereby there as a greater number of errors for target than non-target trials. Furthermore, there was a condition*trial type interaction (*F* (1, 31) = 7.444, *p* = 0.01, *d* = 0.98). Pairwise comparisons indicated errors were greater in the hangover condition than the no-hangover condition for both target (*F* (1, 33) = 21.700, *p* < 0.001, *d* = 1.62) and non-target trials (*F* (1, 33) = 4.454, *p* = 0.042, *d* = 0.74). Furthermore, errors for target trials were greater than errors for non-target trials within both the hangover (*F* (1, 33) = 24.087, *p* < 0.001, *d* = 1.71) and no-hangover conditions (*F* (1, 33) = 19.080, *p* < 0.001, *d* = 1.52). There were no other significant effects or interactions.

For the more difficult 2-back version of the task, there was a main effect of condition (*F* (1, 31) = 20.708, *p* < 0.01, *d* = 1.63), whereby errors were greater in the hangover than the no-hangover condition. There was also a condition*order interaction (*F* (1, 31) = 6.732, *p* = 0.014, *d* = 0.93) that indicated performance significantly improved across testing days for those completing the hangover condition first (*F* (1, 31) = 28.528, *p* < 0.001, *d* = 1.92), whereas there were no significant differences between testing days for those who completed the task for a second time, whilst hungover (*p* = 0.198) (see Figure 4c). Our analysis also indicated that participants who were sober during their first session made a greater number of errors in the no-hangover condition than those who were sober during their second session (F (1, 22) = 12.958, *p* = 0.002, *d* = 1.54). There were no other significant effects or interactions.

### 3.4. Effects of Hangover on Goal Maintenance

Target and non-target trials were analysed separately, to avoid comparing stimuli presented 70% of the time to non-target stimuli, which were presented 10% of the time each [45]. A 2 (condition) * 2 (order) repeated measures ANOVA indicated a main effect of condition only (*F* (1, 29) = 16.643, *p* < 0.001, *d* = 1.52), whereby AX-type trial errors were greater in the hangover than the no-hangover condition.

Errors for non-target trials (BX-, BY-, and AY-type trials) were analysed separately. Increased errors on BX-type trials in the hangover relative to the no-hangover condition are indicative of a shift toward a reactive control style. There was a trend-level main effect of condition (*F* (1, 28) = 3.279, *p* = 0.081, *d* = 0.69) whereby non-target errors tended to be greater in the hangover relative to the no-hangover condition. In addition, there was a main effect of trial type (*F* (1, 31) = 28.829, *p* < 0.001, *d* = 2.84), whereby there were more errors on AY-type relative to BY-type and BX-type trials and more errors on BX-type relative to BY-type trials. There were no other significant effects or interactions.

### 3.5. Subjective Measures

A series of paired-samples t-tests was used to analyse RSME scores for each task. For the switching task, perceived mental effort was greater (*t* (28) = 3.899, *p* = 0.001, *d* = 0.72) in the hangover condition than the no-hangover condition. For the n-back task, perceived mental effort was also greater (*t* (33) = 3.767, *p* = 0.001, *d* = 0.65) in the hangover condition than the no-hangover condition. Furthermore, perceived mental effort for the AX-CPT task was greater (*t* (31) = 2.818, *p* = 0.008, *d* = 0.50) in the hangover condition than the no-hangover condition. There were lower self-efficacy scores in the hangover relative to the no-hangover condition for the switching task (*t* (33) = 5.816, *p* < 0.001, *d* = 1.00). This difference was marginally significant for the AX-CPT task (*p* = 051), but not the n-back task (*p* = 0.384). There were no sex differences in hangover severity (*p* = 0.790) or eBAC (*p* = 0.195).

### 3.6. Correlational Analysis

Bivariate correlational analysis provided no evidence that hangover-severity scores (as measured by the mAHSS; *ps* > 0.178) and self-efficacy scores (*p*s > 0.098) were associated with performance on the switching, n-back, or AX-CPT tasks. Bivariate correlational analysis also provided no evidence that eBAC was related to hangover severity (*p* = 0.229) or task performance (*p*s ≥ 0.161).

## 4. Discussion

This study demonstrated that switching, updating, and goal maintenance are all impaired during an alcohol hangover. Thus, in terms of the unity/diversity model of executive functions [15], all of the core components of executive function appear to be negatively influenced by a hangover. Errors for non-target trial types on the AX-CPT task (i.e., AY-type, BX-type, or BY-type trials) showed a trend toward being greater in the hangover than the no-hangover condition. Moreover, contrary to our hypothesis, there was no evidence that performance on switching, updating, and goal-maintenance tasks was related to hangover severity. There was also no evidence that hangover-related impairments in task performance were related to self-efficacy during switching, updating, and goal-maintenance task performance. However, the participants felt that they needed to expend greater mental effort to complete each task when experiencing a hangover than when not hungover. Furthermore, there was no influence of sex on cognitive performance when hungover for any of the tasks.

In line with a previous naturalistic study of hangovers [30], our results from the switching task indicate that individuals make a greater number of errors, reflective of deficits in task switching, when they are experiencing a hangover, as opposed to when they are not hungover. This suggests that a hangover impairs an individual’s ability to switch attention from one task or mental set to another effectively. Although studies that experimentally induce hangovers often administer lower doses of alcohol than observed in real-life drinking [2], our null results for an effect of a hangover on switch costs are in line with previous experimental research [19]. Therefore, it appears as though individuals may maintain speed of switching, but become less accurate, when experiencing a hangover, as compared to not being hungover. For switching, our results also tentatively indicated an interaction of condition with order, further suggesting a speed-accuracy trade-off. Those completing the hangover condition first appear to sacrifice time (switch costs) to maintain accuracy during the hangover condition, whereas those completing the hangover condition second appear to sacrifice accuracy to maintain speed.

Our results indicate poorer performance on both the 1-back and 2-back versions of the n-back task in the hangover compared to no-hangover condition. This suggests that an individual’s ability to update information in working memory is impaired during a hangover. As the 1-back version of the task is relatively easy and places a comparatively low load on working memory, the current results suggest that participants with a hangover experienced an increased cognitive load, relative to during a non-hungover state. This is in line with previous research suggesting that a hangover reduces the amount of cognitive resources available [18,19], and it is consistent with our results indicating greater mental effort to complete tasks. Although hangover symptoms, such as headache and fatigue, are known to impair an individual’s ability to update information via increased cognitive load [34], our results indicate no evidence of an association between performance on any task and overall hangover-severity scores. This suggests that hangover-related impairments in executive functions are likely due to factors other than simple cognitive interference due to the presence of negative symptoms. For example, it is possible that physical alterations in hangovers, such as dopaminergic or noradrenergic transmission [9,25], or immune effects (indexed via cytokine levels) [52,53], influence cognition [54]. The observed interaction of condition and order tentatively suggests that those completing the hangover condition first appear to have greater improvement in their second session than those completing the no-hangover condition first. This could indicate an expectancy effect, whereby, when the first condition is during a hangover, participants expect their second performance on the task (i.e., when sober) to be greatly improved.

Results from the current study indicate poorer goal maintenance during hangovers, as reflected by a greater number of errors on the core AX trials of the AX-CPT task in the hangover compared to the no-hangover condition. This suggests that an individual’s ability to maintain and manage goals is impaired whilst experiencing a hangover. Goal maintenance is thought to represent the ‘common factor’ of the unity/diversity model, and an important aspect of maintaining goals is inhibitory control [15]. Therefore, impaired goal maintenance during a hangover may contribute toward findings of previous studies of executive functions that have reported impaired prospective memory, semantic verbal fluency [10], working memory, [12], and inhibitory control [5,6,30] during a hangover relative to a no-hangover condition. Contrary to our hypothesis, there was no evidence that participants were biased toward reactive control during a hangover, suggesting participants engaged in proactive control during this task, but were ineffective in doing so (as evidenced by increased errors on the core AX-type trials). However, it is possible that the current study did not have sufficient power to observe effects on reactive control, due to the low number of non-target trials on this task. As goal maintenance is important for many everyday behaviours that rely on executive functions, such as planning, decision making, organising, and other ‘higher-order’ skills, future studies should investigate the influence of hangovers on these processes.

The current results should be viewed in light of the following strengths and limitations. The crossover, within-subjects design could be considered a strength of the current study, because each subject serves as his or her own control. Furthermore, the naturalistic design, although the naturalistic design is limited in its control over alcohol consumption, it can be considered a strength as it involves investigating the impact of real-life drinking, rather than an experimentally induced hangover, which might involve consuming lower levels of alcohol [41]. However, the study is limited in its ability to generalise beyond the narrow demographics of this student population (i.e., to other age groups, education levels, etc.). Another limitation is the use of the Widmark formula, which should be viewed as a rough estimate of alcohol consumption. Future studies should explore directly measuring BAC during the heavy drinking occasion, possibly via wearable technology. Although each task used in this study was chosen to reflect switching, updating, or goal maintenance, these tasks are cognitively complex (i.e., they measure multiple executive and non-executive functions). One technique that could be utilised in future studies, to overcome variability attributable to task stimuli, rather than the respective executive function component, is the adoption of a latent variable approach, which is a statistical technique that can capture common variance across multiple measures (e.g., [55]).

## 5. Conclusions

Results from the current study indicate that all domains of the unity/diversity model of executive functions are negatively affected by alcohol hangover. Executive functions are important cognitive processes which are utilised in everyday behaviours, such as planning, decision-making, and emotion regulation. Thus, impairments in a range of executive functions could have broad implications for a wide variety of everyday activities, including in the workplace. For example, employees who go to work when experiencing a hangover may negatively influence the productivity and working environment of others [13]. Future studies should aim to investigate the impact of hangover-induced executive dysfunction on the performance of everyday tasks.

## Figures and Tables

**Table 1:** Means, standard deviations, and group comparisons for each variable.

Variable	Hangover		No-Hangover		*p*	Effect Size
	M	SD	M	SD		
**Switching Task**						
Switch Cost (ms)	247.83	87.09	208.95	82.27	0.078	*d* = 0.72
Switch Errors	6.01	2.97	4.92	2.68	0.019 *	*d* = 1.08
**n-back Working Memory Task**						
1-back errors	3.92	1.78	2.47	1.31	<0.001 *	*d* = 1.64
2-back errors	6.89	3.12	4.78	1.66	<0.001 *	*d =* 1.63
**AX-CPT Task**						
Target Errors (AX-type trials)	4.48	4.33	1.79	163	<0.001 *	*d* = 1.52
Non-Target Errors	1.39	1.24	1.00	1.23	0.081	*d* = 0.69
**Hangover Severity**						
1-Item Hangover Severity	3.83	1.84	0	0	<0.001 *	*d* = 2.08
mAHSS	2.40	1.31	0.24	0.26	<0.001 *	*d* = 1.72
**Alcohol Consumption**						
Alcohol Units (night before testing)	13.28	5.13	0	0	<0.001 *	*d* = 5.27
eBAC	0.16%	0.08	0	0	<0.001 *	*d =* 4.04
**Subjective Measures**						
RSME Switching	77.27	23.7	58.72	22.78	0.001 *	*d* = 0.72
RSME n-back	76.41	24.22	58.79	20.81	0.001 *	*d* = 0.65
RSME AX-CPT	59.69	23.70	47.41	28.67	0.008 *	*d* = 0.55
Self-efficacy Switching	6.88	2.14	8.76	1.28	< 0.001 *	*d* = 1.00
Self-efficacy n-back	6.31	2.18	6.74	2.5	0.384	*d =* 0.14
Self-efficacy AX-CPT	8.53	1.38	9.06	1.41	0.051	*d =* 0.35

Notes: M, mean; SD, standard deviation; mAHSS, modified Alcohol Hangover Severity Scale; RSME, Rating Scale of Mental Effort; eBAC, estimated Blood Alcohol Concentration; AX-CPT, AX-Continuous Performance Task. The asterisk indicates that the difference between the hangover and no-hangover conditions was significant.

## References

[1] Van Schrojenstein Lantman M., van de Loo A., Mackus M., Verster J. (2017). Development of a definition for the alcohol hangover: Consumer descriptions and expert consensus. Curr. Drug Abus. Rev..

[2] Gunn C., Mackus M., Griffin C., Munafò M.R., Adams S. (2018). A systematic review of the next-day effects of heavy alcohol consumption on cognitive performance. Addiction.

[3] McGee R., Kypri K. (2004). Alcohol-related problems experienced by university students in New Zealand. Aust. N. Z. J. Public Health.

[4] Devenney L.E., Coyle K.B., Verster J.C. (2019). Cognitive performance and mood after a normal night of drinking: A naturalistic alcohol hangover study in a non-student sample. Addict. Behav. Rep..

[5] McKinney A., Coyle K., Penning R., Verster J.C. (2012). Next day effects of naturalistic alcohol consumption on tasks of attention. Hum. Psychopharmacol..

[6] Gunn C., Verster J.C., Adams S. (2019). The effects of alcohol hangover on response inhibition and attentional bias towards alcohol-related stimuli. Alcohol. Clin. Exp. Res..

[7] Noël X., Bechara A., Dan B., Hanak C., Verbanck P. (2007). Response inhibition deficit is involved in poor decision making under risk in nonamnesic individuals with alcoholism. Neuropsychology.

[8] Schmeichel B.J., Volokhov R.N., Demaree H.A. (2008). Working memory capacity and the self-regulation of emotional expression and experience. J. Pers. Soc. Psychol..

[9] Howse A.D., Hassall C.D., Williams C.C., Hajcak G., Krigolson O.E. (2018). Alcohol hangover impacts learning and reward processing within the medial-frontal cortex. Psychophysiology.

[10] Heffernan T., Samuels A., Hamilton C., McGrath-Brookes M. (2019). Alcohol hangover has detrimental impact upon both executive function and prospective memory. Front. Psychiatry.

[11] Heffernan T. (2018). A state of alcohol hangover impedes everyday prospective memory. Front. Hum. Neurosci..

[12] Howland J., Rohsenow D.J., Greece J.A., Littlefield C.A., Almeida A., Heeren T., Winter M., Bliss C.A., Hunt S., Hermos J. (2010). The effects of binge drinking on college students’ next-day academic test-taking performance and mood state. Addiction.

[13] Bhattacharya A. (2019). Financial Headache: The Cost of Workplace Hangovers and Intoxication to the UK Economy.

[14] Husain M. Executive function and behaviour. Proceedings of the 3rd Congress of the European Academy of Neurology.

[15] Friedman N.P., Miyake A. (2017). Unity and diversity of executive functions: Individual differences as a window on cognitive structure. Cortex.

[16] Lépine R., Bernardin S., Barrouillet P. (2005). Attention switching and working memory spans. Eur. J. Cogn. Psychol..

[17] Zink N., Bensmann W., Beste C., Stock A.-K. (2018). Alcohol hangover increases conflict load via faster processing of subliminal information. Front. Hum. Neurosci..

[18] Scholey A., Ayre B., Terpstra C., Benson S. (2019). Alcohol hangover results in reduced attentional resources. Alcohol. Clin. Exp. Res..

[19] Wolff N., Gussek P., Stock A.-K., Beste C. (2016). Effects of high-dose ethanol intoxication and hangover on cognitive flexibility. Addict. Biol..

[20] Eysenck M.W., Derakshan N., Santos R., Calvo M.G. (2007). Anxiety and cognitive performance: Attentional control theory. Emotion.

[21] Lavie N., Dalton P. (2014). Load Theory of Attention and Cognitive Control.

[22] Lavie N., Hirst A., de Fockert J.W., Viding E. (2004). Load theory of selective attention and cognitive control. J. Exp. Psychol. Gen..

[23] Stock A.-K., Beste C. (2014). Binge drinking and the differential influence of ethanol on cognitive control subprocesses: A novel field of neurotoxicology. Arch. Toxicol..

[24] Goldstein R.Z., Volkow N.D. (2011). Dysfunction of the prefrontal cortex in addiction: Neuroimaging findings and clinical implications. Nat. Rev. Neurosci..

[25] Maki T., Toivonen L., Koskinen P., Naveri H., Harkonen M., Leinonen H. (1998). Effect of ethanol drinking, hangover, and exercise on adrenergic activity and heart rate variability in patients with a history of alcohol-induced atrial fibrillation. Am. J. Cardiol..

[26] Penning R., McKinney A., Verster J.C. (2012). Alcohol hangover symptoms and their contribution to the overall hangover severity. Alcohol Alcohol..

[27] Van Der Linden D., Frese M., Meijman T.F. (2003). Mental fatigue and the control of cognitive processes: Effects on perseveration and planning. Acta Psychol. (Amst).

[28] Verster J.C., de Klerk S., Bervoets A.C., Kruisselbrink L.D. (2014). Editorial: Can hangover immunity be really claimed?. Curr. Drug Abus. Rev..

[29] Scholey A., Benson S., Kaufman J., Terpstra C., Ayre E., Verster J.C., Allen C., Devilly G. (2019). Effects of alcohol hangover on cognitive performance: Findings from a field/internet mixed methodology study. J. Clin. Med..

[30] Devenney L.E., Coyle K.B., Verster J.C. (2019). Memory and attention during an alcohol hangover. Hum. Psychopharmacol..

[31] Speer N.K., Jacoby L.L., Braver T.S. (2003). Strategy-dependent changes in memory: Effects on behavior and brain activity. Cogn. Affect. Behav. Neurosci..

[32] Gonthier C., Macnamara B.N., Chow M., Conway A.R.A., Braver T.S. (2016). Inducing proactive control shifts in the AX-CPT. Front. Psychol..

[33] Botto M., Basso D., Ferrari M., Palladino P. (2014). When working memory updating requires updating: Analysis of serial position in a running memory task. Acta Psychol. (Amst).

[34] Moore D.J., Keogh E., Eccleston C. (2013). Headache impairs attentional performance. Pain.

[35] Moore D.J., Eccleston C., Keogh E. (2017). Cognitive load selectively influences the interruptive effect of pain on attention. Pain.

[36] Martínez-Cancino D.P., Azpiroz-Leehan J., Jiménez-Angeles L. (2015). The Effects of Sleep Deprivation in Working Memory Using the N-Back Task.

[37] Rohsenow D.J., Howland J., Arnedt J.T., Almeida A.B., Greece J., Minsky S., Kempler C.S., Sales S. (2010). Intoxication with Bourbon versus Vodka: Effects on hangover sleep and next-day neurocognitive performance in young adults. Alcohol. Clin. Exp. Res..

[38] Verster J.C. (2007). Sleep after an evening of heavy drinking and its impact on daytime sleepiness and alcohol hangover severity. Sleep Biol. Rhythm..

[39] Chow J.T., Hui C.M., Lau S. (2015). A depleted mind feels inefficacious: Ego-depletion reduces self-efficacy to exert further self-control. Eur. J. Soc. Psychol..

[40] Finnigan F., Schulze D., Smallwood J., Helander A. (2005). The effects of self-administered alcohol-induced “hangover” in a naturalistic setting on psychomotor and cognitive performance and subjective state. Addiction.

[41] Verster J.C., Van De Loo A.J., Adams S., Stock A., Benson S., Scholey A., Alford C., Bruce G. (2019). Advantages and limitations of naturalistic study designs and their implementation in alcohol hangover research. J. Clin. Med..

[42] Monsell S., Sumner P., Waters H. (2003). Task-set reconfiguration with predictable and unpredictable task switches. Mem. Cogn..

[43] Attridge N., Eccleston C., Noonan D., Wainwright E., Keogh E. (2017). Headache impairs attentional performance: A conceptual replication and extension. J. Pain.

[44] Braver T.S., Rush B.K., Satpute A.B., Racine C.A., Barch D.M. (2005). Context processing and context maintenance in healthy aging and early stage dementia of the Alzheimer’s type. Psychol. Aging.

[45] Paxton J.L., Barch D.M., Racine C.A., Braver T.S. (2008). Cognitive control, goal maintenance, and prefrontal function in healthy aging. Cereb. Cortex.

[46] Kypri K., Langley J., Stephenson S. (2005). Episode-centred analysis of drinking to intoxication in university students. Alcohol Alcohol..

[47] Hogewoning A., Van de Loo A., Mackus M., Raasveld S.J., De Zeeuw R., Bosma E., Bouwmeester N., Brrokhuis K.A., Garssen J., Verster J.C. (2016). Characteristics of social drinkers with and without a hangover after heavy alcohol consumption. Subst. Abus. Rehabil..

[48] Zijlstra F.R., Van Doorn L. (1985). The Construction of a Scale to Measure Perceived Effort.

[49] Gunn C., Fairchild G., Verster J., Adams S. The Effects of Alcohol Hangover on Executive Functions osf.io/n3ydu. https://osf.io/n3ydu.

[50] Longman C.S., Lavric A., Monsell S. (2016). The coupling between spatial attention and other components of task-set: A task-switching investigation. Q. J. Exp. Psychol..

[51] Holloway F.A. (1994). Low-Dose Alcohol Effects on Human Behavior and Performance: A Review of Post-1984 Research.

[52] Van de Loo A., Hogewoning A., Raasveld S.J., De Zeeuw R., Bosma E.R., Bouwmeester N.H., Lukkes M., Brookhuis K.A., Knipping K., Garssen J. (2015). Saliva cytokine concentrations the day after heavy alcohol consumption in drinkers suffering from a hangover versus those who claim to be hangover resistant. Alcohol Alcohol..

[53] Kim D.-J., Kim W., Yoon S.-J., Choi B.-M., Kim J.-S., Go H.J., Kim Y.-K., Jeong J. (2003). Effects of alcohol hangover on cytokine production in healthy subjects. Alcohol.

[54] Tipple C.T., Benson S., Scholey A. (2017). A review of the physiological factors associated with alcohol hangover. Curr. Drug Abus. Rev..

[55] Korucuoglu O., Sher K.J., Wood P.K., Saults J.S., Altamirano L., Miyake A., Bartholow B.D. (2017). Acute alcohol effects on set-shifting and its moderation by baseline individual differences: A latent variable analysis. Addiction.

